# Supplementary material for: Allosteric gate modulation confers K+ coupling in glutamate transporters
Source: EMBO J. 2019 Sep 10;38(19):e101468. doi: 10.15252/embj.2019101468 (PMC6769379; doi:10.15252/embj.2019101468)
Supplement: Supplementary file 1 — Appendix [file EMBJ-38-e101468-s001.pdf]

Appendix

Table of contens:

Figure S1	p2
Figure S2	p4
Figure S3	p6
Figure S4	p8
Figure S5	p10
Figure S6	p11
Figure S7	p13
Figure S8	p15
Figure S9	p17
Table S1	p19
Table S2	p19
Table S3	p20
Table S4	p20
Table S5	p20
Table S6	p21

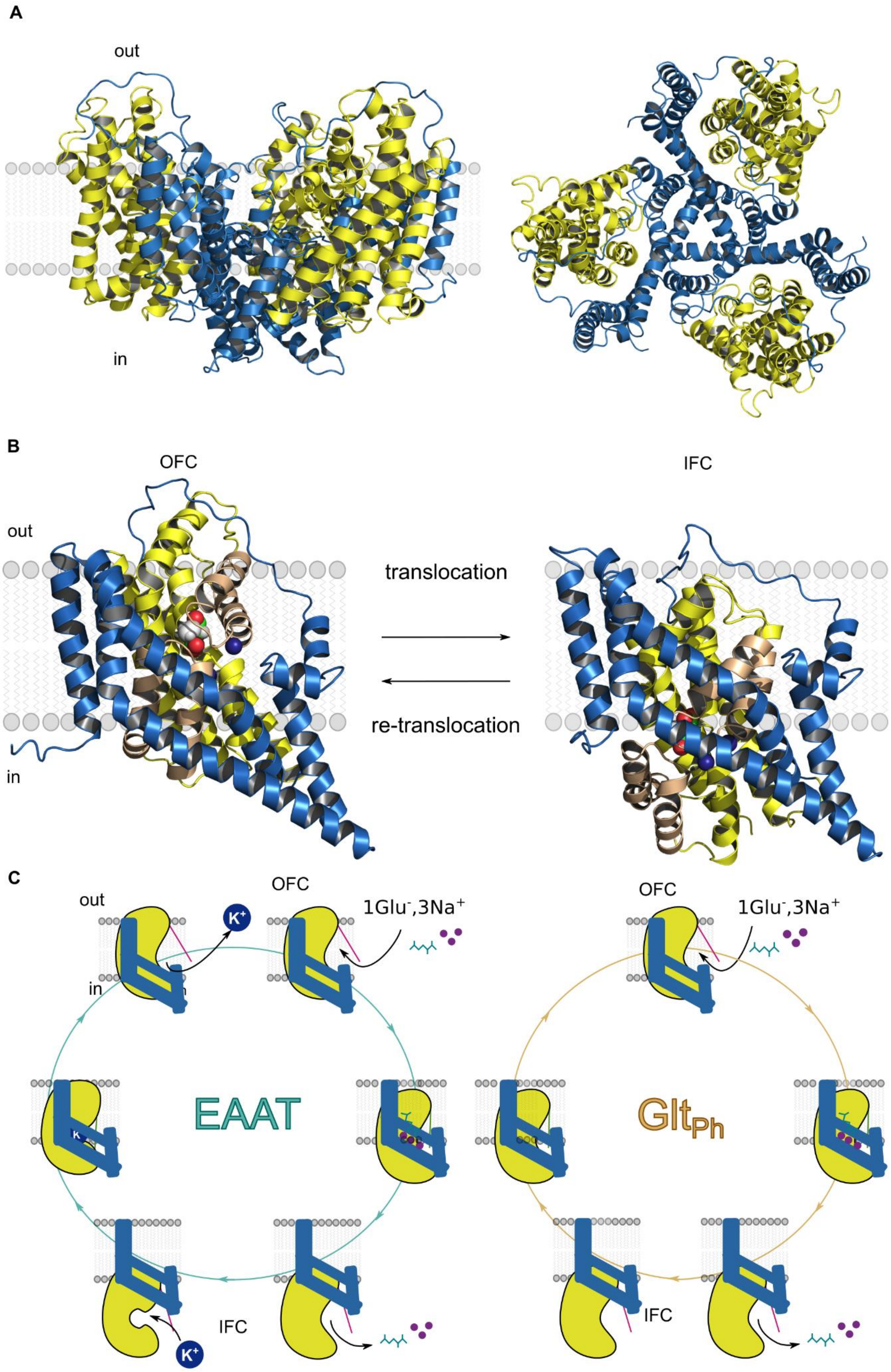
A
out
in
B
OFC
IFC
out
translocation
re-translocation
in
C
out
OFC
1Glu-,3Na+
K+
in
EAAT
IFC
K+
OFC
1Glu-,3Na+
GltPh
IFC

Appendix Figure S1. Structure and function of EAATs/Glt$_X$.

(A) Trimeric structure of Glt$_{Ph}$ (PDB ID 2NWX; left, side view from within the membrane indicated by cartoon lipids; right, view from the intracellular side). The trimerization domain is colored in blue, the transport domain in yellow.

(B) OFC–IFC transition in Glt$_{Ph}$. The left panel shows the transport domain facing to the extracellular side (PDB ID 2NWX). During substrate translocation, this domain performs an elevator-like movement across the membrane towards the intracellular side (right panel; PDB ID 3KBC).

(C) Cartoon representation of EAAT (left) and Glt$_X$ (right) transport cycles. In both transporters, the cycle starts with substrate binding from the extracellular solution, followed by closure of the HP2 gate and the transport domain translocation to reach the IFC. In Glt$_{Ph}$ the transport domain re-translocates in its apo form, whereas in EAATs this transition is K^+ dependent.

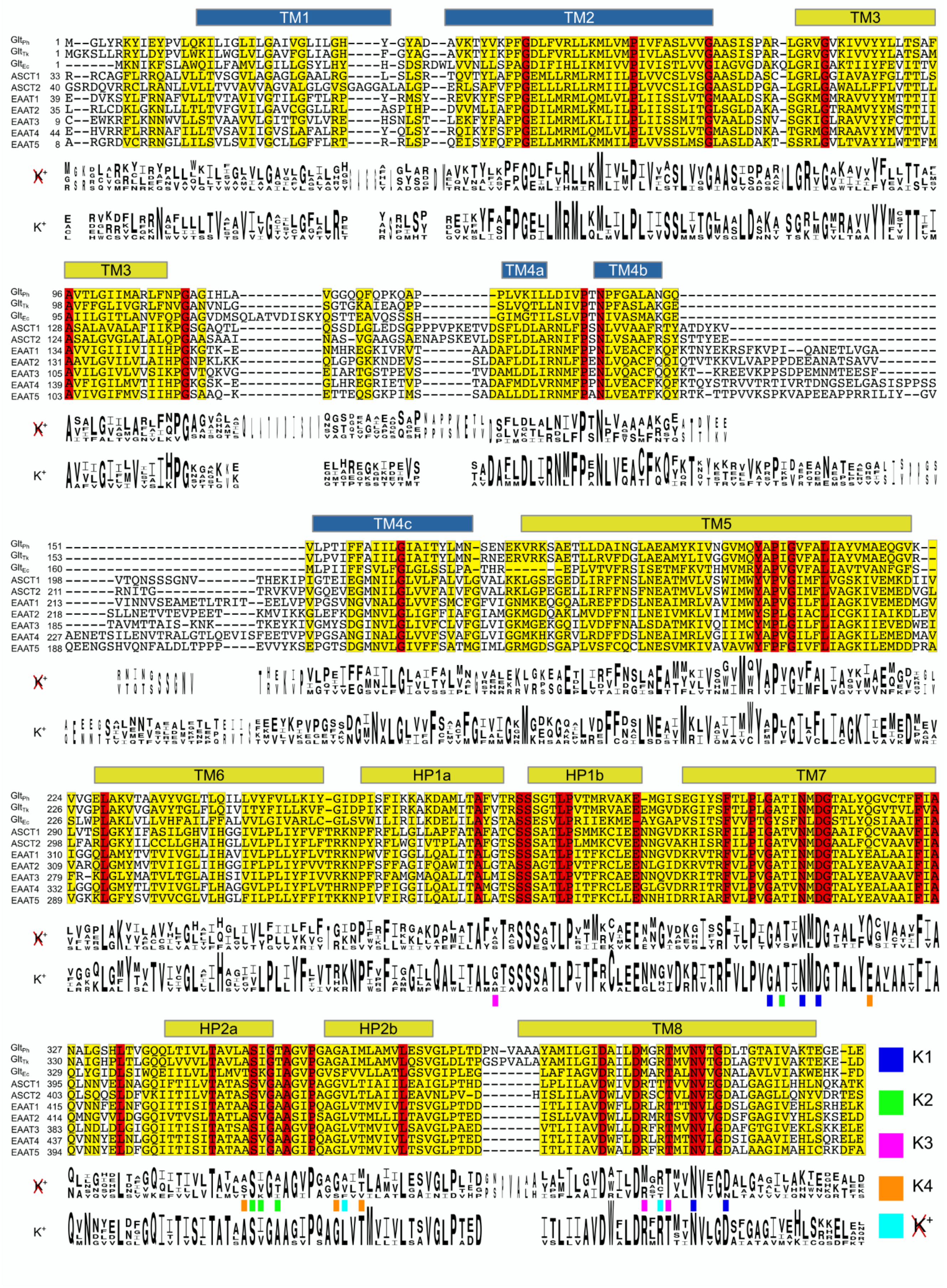
TM1
TM2
TM3
TM4a
TM4b
TM4c
TM5
TM6
HP1a
HP1b
TM7
HP2a
HP2b
TM8
GltPh
GltTk
GltEc
ASCT1
ASCT2
EAAT1
EAAT2
EAAT3
EAAT4
EAAT5
K+
K1
K2
K3
K4

Appendix Figure S2. Sequence comparison of K^+-dependent and independent transporters.

Sequence alignment of Glt_X, Glt_{Ec}, ASCT1/2 and EAAT1–5. Sequence conservation is illustrated as separate sequence logos for K^+-dependent (Glt_X, Glt_{Ec}, ASCT1/2) and K^+-independent (EAAT1–5) transporters.

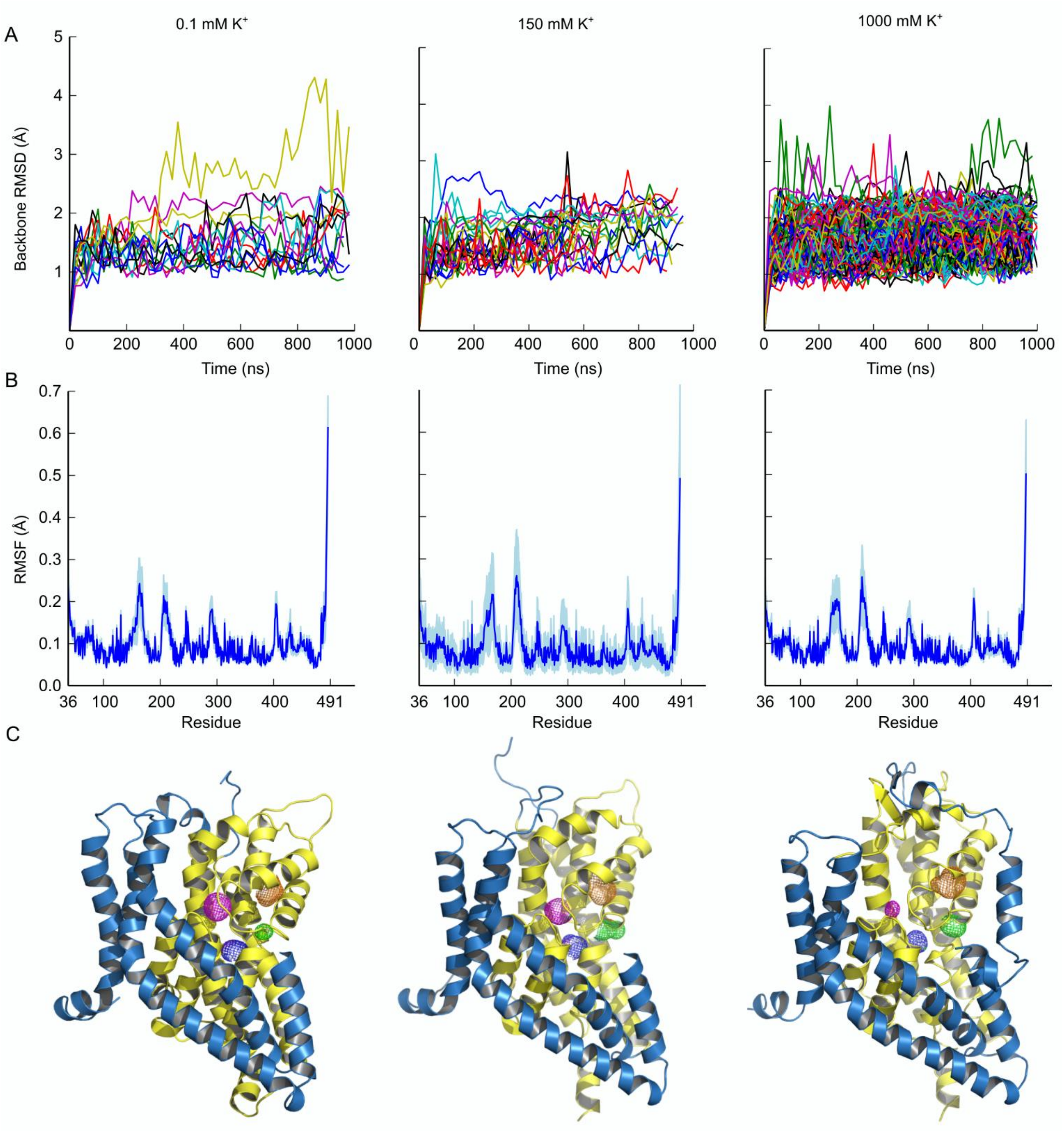

Appendix Figure S3. Protein dynamics and K^+ binding sites and are robust against a large range of $[K^+]$.

(A) Protein backbone root-mean-square deviation (RMSD) for different bulk $[K^+]$. Plots show the time course of the backbone RMSD of the transport domain without HP2 with respect to the starting structure after fitting to the starting structure. We focused on the transport domain because all substrate binding sites are known to be located in this domain.

(B) Per-residue-averaged RMSFs for different [K^+] (defined in A).

(C) Density maps for different [K^+] (defined in A). Isodensity meshes illustrate the K^+ distribution (contoured at 3.5 σ) around EAAT1 monomers in the OFC at different K^+ concentrations.

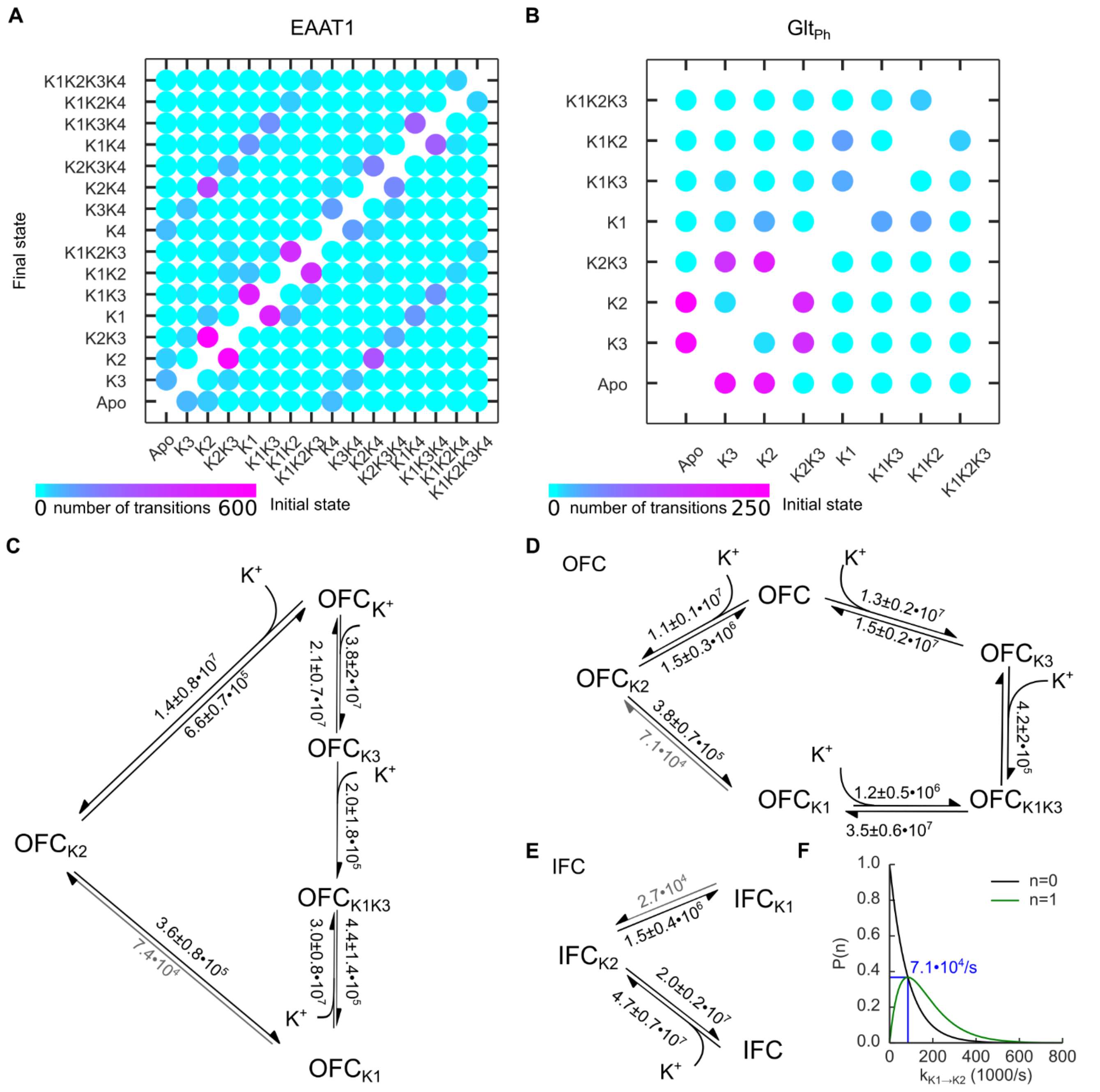

Appendix Figure S4. Kinetic parameters of K^+-binding transitions.

(A,B) Transition density plots for EAAT1 (A) and Glt_{Ph} OFC (B). Colors indicate total numbers of transitions observed in unguided MD simulations.

(C,D,E) Calculated rates (s^{-1}) of K^+ binding/unbinding pathways with highest reactive fluxes for EAAT1 (C) and Glt_{Ph} (D,E). Values represent the mean ± SD obtained from 1000 bootstrap samples.

(E) Estimation of a lower limit for the OFC_{K1}–OFC_{K2} transition rate for Glt_{Ph} OFC.

(F). The probabilities of observing either none or one K^+-relocation event in the total simulation time spent in K1-bound states are plotted against the transition rate. The calculated rate for one instead of no unbinding event (blue vertical line) corresponds to a probability of 37% of observing no unbinding event.

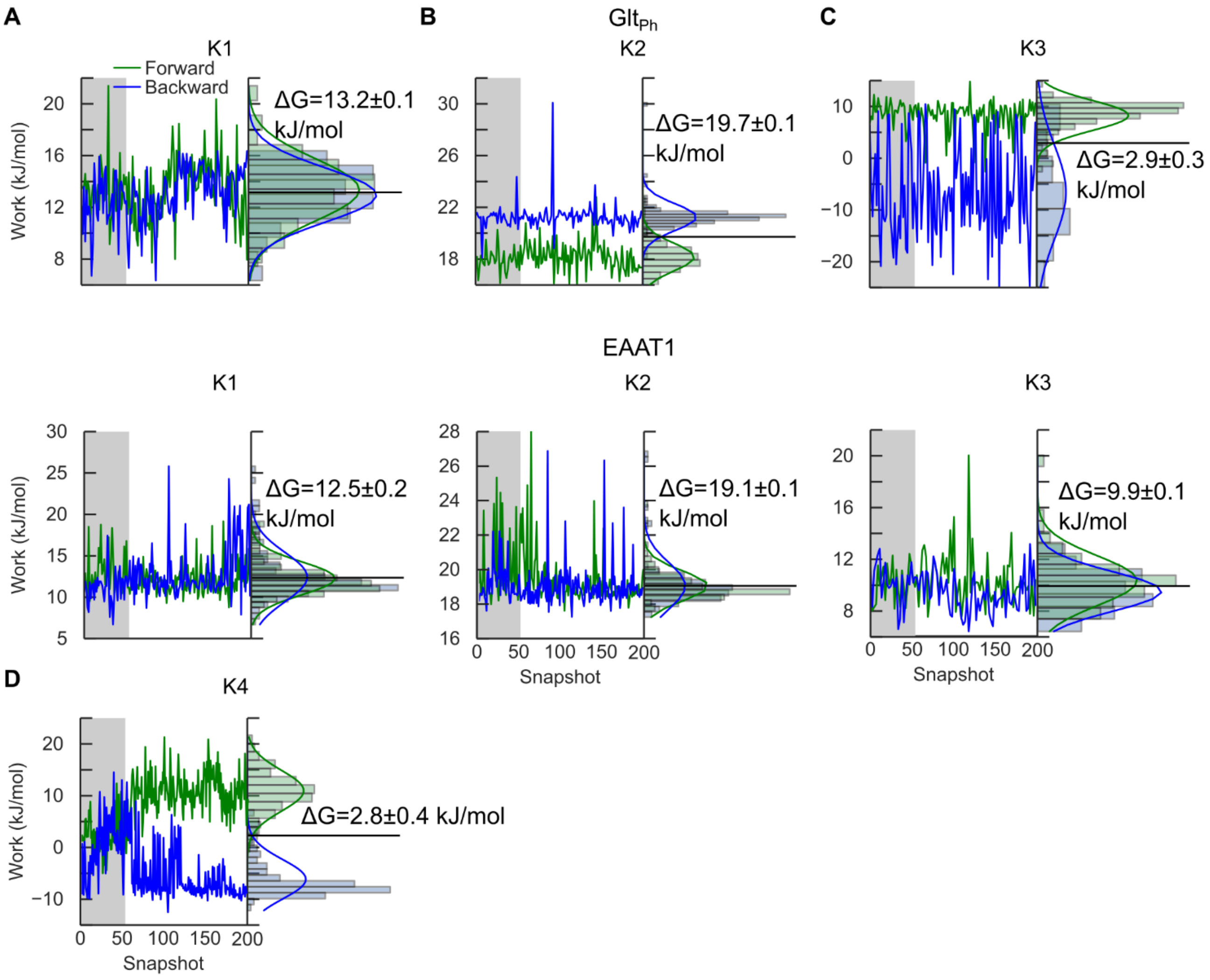

Appendix Figure S5. Simulated Na^+/K^+-binding selectivities in Glt_{Ph} and EAAT1.

Work performed in individual alchemical switching simulations plotted vs the snapshot number, from which the switching was started. Forward switching converts K^+ ions bound to K1 (A), K2 (B), K3 (C) or K4 (D) to Na^+, and backward switching is the reverse process. The right panel shows the resulting work distributions. Data from the gray area was discarded as equilibration and not used in the calculation of the work distributions. The solid line indicates the change in free energy, as estimated by the Crooks Gaussian intersection method (Gapsys et al., 2015; Goette & Grubmüller, 2009).

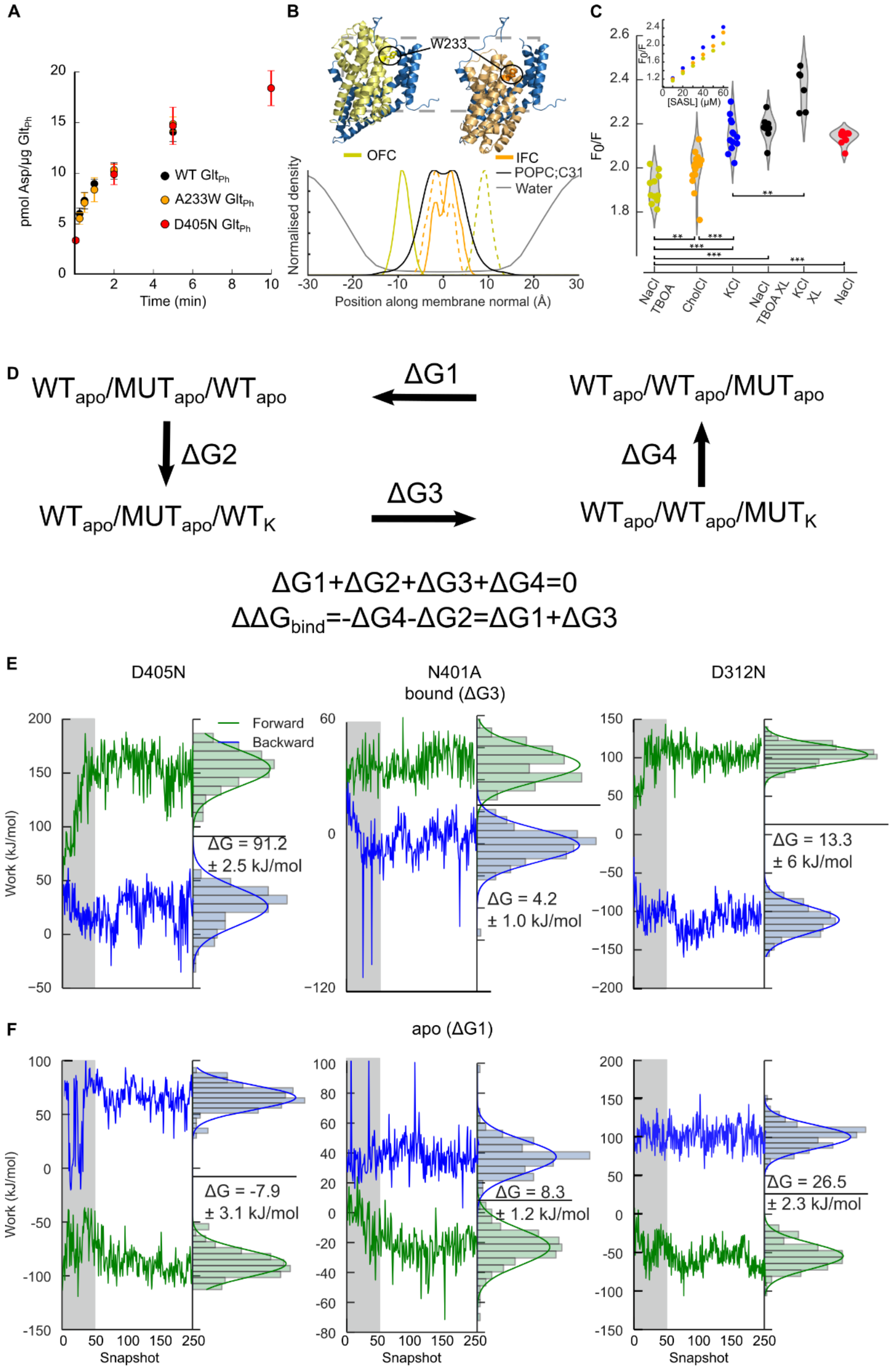
A
B
C
D
E
F
pmol Asp/µg Glt_{Ph}
Time (min)
WT Glt_{Ph}
A233W Glt_{Ph}
D405N Glt_{Ph}
W233
OFC
IFC
POPC;C31
Water
Normalised density
Position along membrane normal (Å)
F_0/F
[SASL] (µM)
NaCl TBOA
CholCl
KCl
NaCl TBOA XL
KCl XL
NaCl
$WT_{apo}/MUT_{apo}/WT_{apo}$
ΔG1
$WT_{apo}/WT_{apo}/MUT_{apo}$
ΔG2
ΔG4
$WT_{apo}/MUT_{apo}/WT_K$
ΔG3
$WT_{apo}/WT_{apo}/MUT_K$
$\Delta G1+\Delta G2+\Delta G3+\Delta G4=0$
$\Delta\Delta G_{bind}=-\Delta G4-\Delta G2=\Delta G1+\Delta G3$
D405N
N401A
bound (ΔG3)
D312N
Forward
Backward
Work (kJ/mol)
ΔG = 91.2 ± 2.5 kJ/mol
ΔG = 4.2 ± 1.0 kJ/mol
ΔG = 13.3 ± 6 kJ/mol
apo (ΔG1)
ΔG = -7.9 ± 3.1 kJ/mol
ΔG = 8.3 ± 1.2 kJ/mol
ΔG = 26.5 ± 2.3 kJ/mol
Snapshot

Appendix Figure S6. Mutation-induced free-energy changes for K^+ binding.

(A) Aspartate uptake by WT, D405N and A233W Glt_{Ph} (EAAT1 D487, V319). Time-dependent uptake of radiolabeled aspartate into liposomes normalized to the protein amount (mean ± SD, 4 experiments).

(B) Position of residue 233 in Glt_{Ph} in OFC and IFC. Density profiles of residue 233 in MD simulations. Profiles are mirrored (dotted lines) to account for random orientation of Glt_{Ph} in the liposome membrane (Ryan et al., 2009). Position of the quencher is approximated by the C31 atom of a POPC molecule (Subczynski et al., 2009).

(C) Violin plots of the F_0/F distribution for buffers containing NaCl+TBOA, CholCl, KCl or NaCl for WT protein or NaCl+TBOA or KCl for cross-linked protein (XL). Scatter plots show individual measurements. Statistical significance was tested by two-tailed unpaired t-test (**$p<0.01$, ***$p<0.001$). Inset shows representative [16-SASL] dependences of tryptophan fluorescence of A233W Glt_{Ph} in different buffers.

(D) The thermodynamic cycle used for calculating mutation-induced free-energy changes for K^+ binding to K1 with indicated compositions of the Glt_{Ph} trimer. We simulated the horizontal transitions, i.e. transforming a WT monomer into a mutant (defined in B) and vice versa to obtain the free-energy differences between the vertical transitions as shown in the equation.

(E,F) Work performed in individual alchemical switching simulations for calculating $\Delta G3$ (B) or $\Delta G1$ (C) plotted against the snapshot from which the switching was started; resulting work distributions are shown on the right. Data from the gray area was discarded as equilibration and not used in the calculation of the work distributions. The solid line indicates the change in free energy, as estimated by the Crooks Gaussian intersection method (Gapsys et al., 2015; Goette & Grubmüller, 2009).

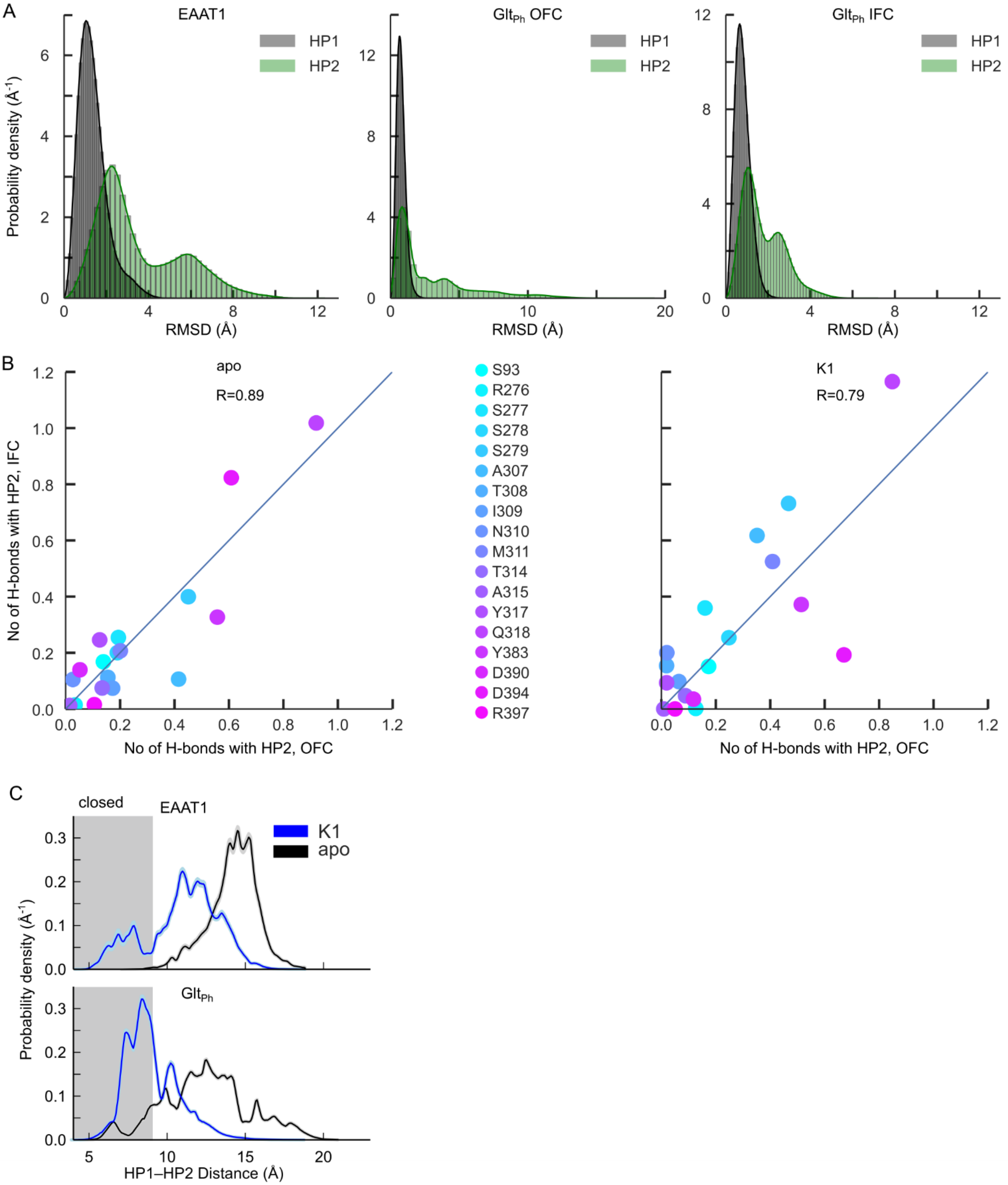

Appendix Figure S7. HP2 dynamics and contacts with the transport domain are identical in the outward- and inward-facing states.

(A) Root-mean-square deviation of the tips of HP1 and HP2 with respect to their closed conformations in all unguided MD simulations.

(B) Correlation of hydrogen bond numbers between transport domain residues and HP2 in simulations of OFC and IFC Glt_{Ph} with closed HP2.

(C) Probability density distribution for HP2 opening in EAAT1 and OFC Glt_{Ph} from umbrella sampling simulations.

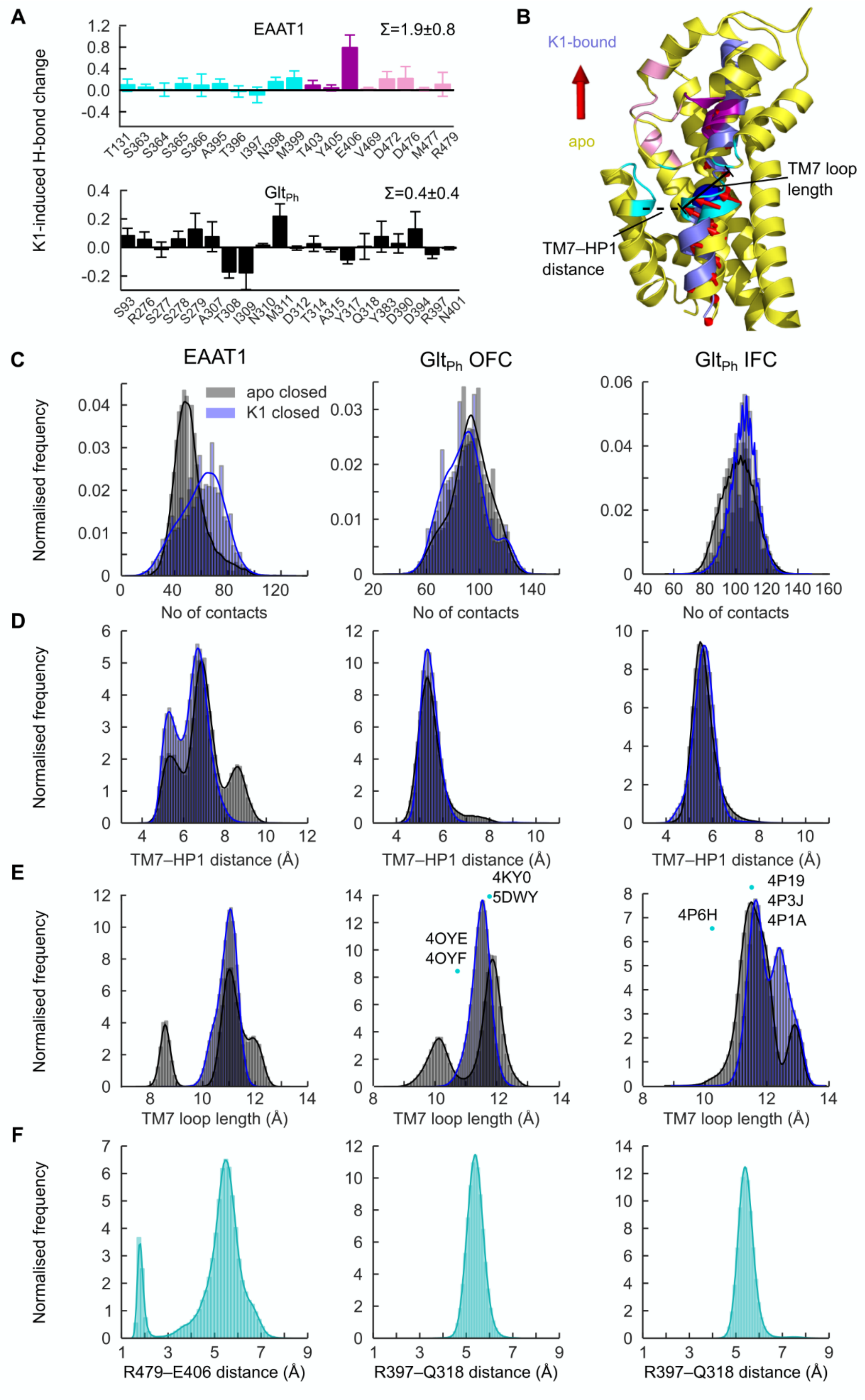
A
K1-induced H-bond change
EAAT1
Σ=1.9±0.8
T131 S363 S364 S365 S366 A395 T396 I397 N398 M399 T403 Y405 E406 V469 D472 D476 M477 R479
Glt$_{Ph}$
Σ=0.4±0.4
S93 R276 S277 S278 S279 A307 T308 I309 N310 M311 D312 T314 A315 Y317 Q318 Y383 D390 D394 R397 N401
B
K1-bound
apo
TM7 loop length
TM7–HP1 distance
C
EAAT1
Glt$_{Ph}$ OFC
Glt$_{Ph}$ IFC
apo closed
K1 closed
Normalised frequency
No of contacts
D
TM7–HP1 distance (Å)
E
TM7 loop length (Å)
4KY0
5DWY
4OYE
4OYF
4P19
4P3J
4P1A
4P6H
F
R479–E406 distance (Å)
R397–Q318 distance (Å)

Appendix Figure S8. Conformational changes induced by K1 occupation.

(A) Changes in the number of hydrogen bonds between the transport domain and HP2 upon K1 occupation. Bars are colored according to the regions highlighted in (B). The sum of all values represents the net change in hydrogen bond number between HP2 and the rest of the transport domain. Values are given as mean ± SD from 1000 bootstrap samples. To analyze the effect of K1 binding on the transport domain, we first measured the HP1–HP2 distance and the K^+ occupation state in every frame of our simulations. We then selected frames that either are K1 bound (with the other sites not occupied) with closed HP gate or in the apo state with closed HP2 gate. C–E show distributions when analyzing those data sets separately.

(B) To visualize the conformational changes in the EAAT1 transport domain induced by K^+-binding to K1, we extracted a representative snapshot in the apo state with high TM7–HP1 distance and another one after occupation of K1 with low distance according to the peaks in the TM7–HP1 distance distributions shown in (D). Overlay of TM7 before (yellow cartoon) and after (blue) K1 occupation; regions with a change in hydrogen bond number are colored according to A.

(C) Contact number distribution (where a contact is defined as an atom pair with a distance <3 Å) between the tip of HP2 and the transport domain for HP2-closed apo and HP2-closed K1-bound states.

(D) TM7–HP1 distance (defined as the distance of the C_α atoms of residues 366 and 395 in EAAT1, 279 and 307 in Glt_{Ph},, dotted black line in B) distributions for HP2-closed apo and HP2-closed K1-bound states.

(E) Length distribution for the unwound part of TM7 (distance between the C_α atoms of residues 395 and 400 in EAAT1; residues 307 and 312 in Glt_{Ph}, solid black line in B) for HP2-closed apo and HP2-closed K1-bound states. Circles in the plot indicate values measured in Glt_{Ph} crystal structures labeled with their PDB codes.

(F) E406–R479 and Q318–R397 distance distributions in simulations of EAAT1, OFC and IFC Glt_{Ph} as observed in all unguided simulations.

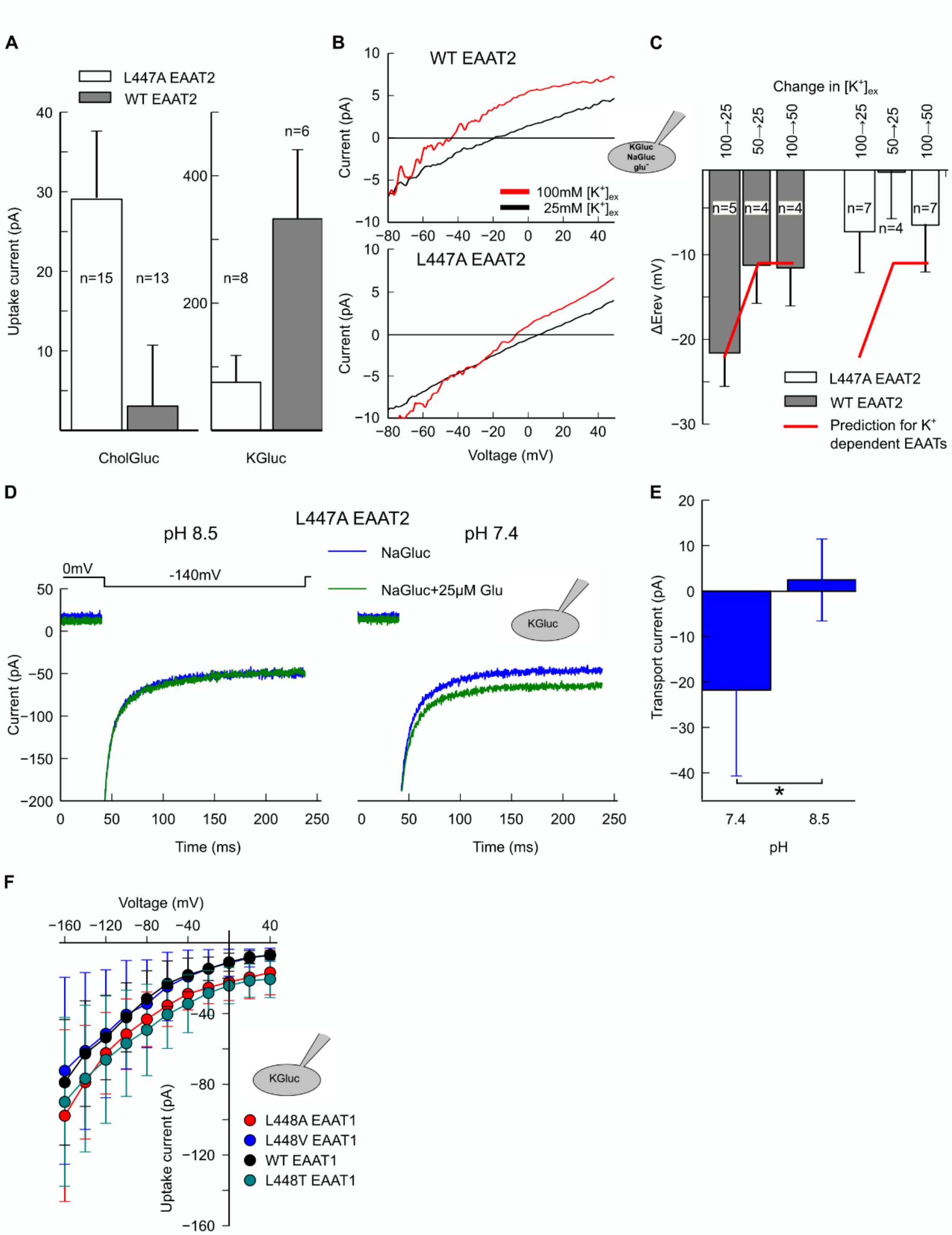

Appendix Figure S9.

(A) Net transport current amplitudes for WT and L447A EAAT2 (Glt_{Ph} A360) for different pipette solutions. Values are given as mean ± SD with indicated numbers of experiments.

(B) Mean TBOA sensitive transport currents for different [K^+] for WT (n=5) and L447A EAAT2 (n=7).

(C) Shift in reversal potential (ΔE_{rev}) for changes in external [K^+]. Values are given as mean ± SD for the indicated number of experiments. Red line shows theoretical predictions for ΔE_{rev} from the zero flux equation

$$E_{rev} = \frac{\mathrm{RT}}{3\mathrm{F}} \cdot \ln\left(\frac{[\mathrm{Na}]_o^3}{[\mathrm{Na}]_i^3} \cdot \frac{[\mathrm{K}]_i}{[\mathrm{K}]_o} \cdot \frac{[\mathrm{H}]_o}{[\mathrm{H}]_i} \cdot \frac{[\mathrm{Glu}]_o}{[\mathrm{Glu}]_i}\right)$$

(Zerangue & Kavanaugh, 1996).

(D) Representative transport-current measurements for a HEK293T cell expressing L447A EAAT2. Cells were dialyzed with Kgluconate-based solutions, and perfusion with a Nagluconate-based external solution with or without 25 µM Glu (Tao & Grewer Biochemistry). Transport currents are calculated by subtracting current amplitudes obtained with glutamate from currents measured in the abscence of glutamate.

(E) L447A EAAT2 uptake current amplitudes at external pH 7.4 or pH 8.5 (mean ± SD, 4 experiments).

(F) Current-voltage relationship for net transport current amplitudes for WT and L448A/T/V EAAT1 (Glt_{Ph} A360) for K^+-based pipette solutions (mean ± SD, 4 experiments).

Appendix Table S1. Related to Figure1. Overview of simulation systems.

No. of K^+	Bulk $[K^+]$ (mM)	No. of POPC	No of Water	Box size ($Å^3$)	No. of replicas	Range of trajectory lengths (ns)	Total length (µs)
**EAAT1 simulations (PDB ID 5LLU)**							
6	0.1	526	57697	144×144×124	5	960–985	4.85
150	130	526	51599	142×142×120	5	915–1062	5.1
953	1000	526	51599	141×141×124	103	262–1150	83.6
**OFC Glt_{Ph} simulations (PDB ID 2NWX)**							
9	0.1	528	45518	141×141×110	5	1008–1222	5.9
856	1000	528	45518	141×141×112	79	190–1390	70.9
**IFC Glt_{Ph} simulations (PDB ID 3KBC)**							
9	0.1	538	45550	142×142×110	5	740–778	3.8
858	1000	538	45550	142×142×112	75	94–1080	54.4

Appendix Table S2. Related to Figure 2. Number of transitions in Glt_{Ph} simulations.

	apo	K3	K2	K2K3	K1	K1K3	K1K2	K1K2K3
**OFC**								
**apo**	408897	495	306	0	2	0	0	0
**K3**	489	420344	21	87	6	4	0	0
**K2**	94	40	2573872	386	37	0	1	0
**K2K3**	0	106	346	236758	0	1	2	0
**K1**	0	0	0	0	505168	118	32	0
**K1K3**	0	0	0	0	117	45540	0	1
**K1K2**	0	0	0	0	30	0	57319	24
**K1K2K3**	0	0	0	0	0	1	24	8609
**IFC**								

**apo**	640893	30	1025	0	3	0	0	0
**K3**	19	137095	3	183	2	0	0	0
**K2**	854	1	1269057	31	92	0	6	0
**K2K3**	0	190	20	67311	0	0	0	0
**K1**	0	0	2	0	686515	1	198	0
**K1K3**	0	0	0	0	1	8	0	0
**K1K2**	0	0	0	0	166	0	454906	0
**K1K2K3**	0	0	0	0	0	0	0	0

Appendix Table S3. Related to Figure 2. Number of transitions in EAAT1 simulations.

	**apo**	**K3**	**K2**	**K3K2**	**K1**	**K1K3**	**K1K2**	**K1K2K3**	**K4**	**K3K4**	**K2K4**	**K2K3K4**	**K1K4**	**K1K3K4**	**K1K2K4**	**K1K2K3K4**
**apo**	36313	190	10	0	0	0	0	0	134	0	0	0	0	0	0	0
**K3**	191	116054	0	16	1	7	0	0	1	451	0	0	0	0	0	0
**K2**	21	2	2574227	966	45	0	33	0	0	0	2602	1	0	0	0	0
**K3K2**	0	26	910	432896	0	6	3	8	0	2	3	815	0	0	0	0
**K1**	0	0	1	0	415187	875	23	0	0	0	0	0	669	4	0	0
**K1K3**	0	1	0	0	857	263678	1	3	0	1	0	0	1	378	0	0
**K1K2**	0	0	1	0	30	0	304525	547	0	0	0	0	0	0	213	0
**K1K2K3**	0	0	0	0	0	7	540	69447	0	0	0	0	0	0	0	46
**K4**	124	1	1	0	0	0	0	0	21137	97	0	0	3	0	0	0
**K3K4**	1	457	0	0	0	0	0	0	86	59599	0	1	1	2	0	0
**K2K4**	0	0	2599	2	0	0	0	0	4	2	333857	148	10	0	1	0
**K2K3K4**	0	0	2	818	0	0	0	0	0	2	135	66990	0	2	0	0
**K1K4**	0	0	0	0	665	3	0	0	0	0	0	0	209864	196	2	0
**K1K3K4**	0	0	0	0	0	369	0	0	0	0	0	0	200	69676	0	0
**K1K2K4**	0	0	0	0	0	0	213	0	0	0	0	0	3	0	7090	8
**K1K2K3K4**	0	0	0	0	0	0	0	43	0	0	0	0	0	0	9	1264

Appendix Table S4. Related to Figure 2. Accumulated dwell-times in Glt_{Ph} simulations (in s).

**OFC**	$2.0\cdot10^{-5}$	$2.1\cdot10^{-5}$	$1.3\cdot10^{-4}$	$1.2\cdot10^{-5}$	$2.5\cdot10^{-5}$	$2.3\cdot10^{-6}$	$2.9\cdot10^{-6}$	$4.3\cdot10^{-7}$
**IFC**	$3.2\cdot10^{-5}$	$6.9\cdot10^{-6}$	$6.4\cdot10^{-5}$	$3.4\cdot10^{-6}$	$3.4\cdot10^{-5}$	$4.5\cdot10^{-10}$	$2.3\cdot10^{-5}$	0.0

Appendix Table S5. Related to Figure 2. Accumulated dwell-times in EAAT1 simulations (in s).

Apo	K3	K2	K2K3	K1	K1K3	K1K2	K1K2K3	K4	K3K4	K2K4	K2K3K4	K1K3	K1K3K4	K1K2K4	K1K2K3K4
$1.83\cdot10^{-6}$	$5.83\cdot10^{-6}$	$1.28\cdot10^{-4}$	2.17e-5	$2.10\cdot10^{-5}$	$1.3\cdot10^{-5}$	$1.32\cdot10^{-5}$	$1.5\cdot10^{-5}$	$3.50\cdot10^{-6}$	$1.1\cdot10^{-6}$	$3.0\cdot10^{-6}$	$1.7\cdot10^{-5}$	$3.4\cdot10^{-6}$	$1.1\cdot10^{-2}$	$3.5\cdot10^{-6}$	$3.7\cdot10^{-7}$

Appendix Table S6. Related to Figure 6. Overview of transport current measurements.

Construct	I_K (pA)	$I_{Choline}$ (pA)	$n_K/n_{Choline}$
I425Q EAAT2	80 ± 11	5.2 ± 1.5	3/3
S429V EAAT2	127 ± 93	1.7 ± 2.0	5/5
T433V EAAT2	114.0 ± 79.5	12 ± 13	4/4
A439T EAAT2	1.4 ± 4.0	1.7 ± 1.2	4/4
S441G S444G EAAT2	36 ± 11	0.8 ± 0.3	4/4
T449M EAAT2	22.8 ± 4.8	0.5 ± 2.2	4/5
M450L EAAT2	294 ± 199	10.5 ± 12	4/5
A362R R476M EAAT2	35 ± 18	2.2 ± 3.1	5/7
V448I EAAT2	127 ± 36	3.1 ± 2.5	3/3
D475S T479A EAAT2	1.6 ± 8.2	11.8± 24	6/6
